# High-angle deflection of metagrating-integrated laser emission for high-contrast microscopy

**DOI:** 10.1038/s41377-023-01286-0

**Published:** 2023-10-13

**Authors:** Mindaugas Juodėnas, Erik Strandberg, Alexander Grabowski, Johan Gustavsson, Hana Šípová-Jungová, Anders Larsson, Mikael Käll

**Affiliations:** 1 Department of Physics, Chalmers University of Technology, 412 96 Gothenburg, Sweden; 2 Department of Microtechnology and Nanoscience, Chalmers University of Technology, 412 96 Gothenburg, Sweden

**Keywords:** Metamaterials, Total internal reflection microscopy, Nanophotonics and plasmonics, Semiconductor lasers, Integrated optics

## Abstract

Flat metaoptics components are looking to replace classical optics elements and could lead to extremely compact biophotonics devices if integrated with on-chip light sources and detectors. However, using metasurfaces to shape light into wide angular range wavefronts with high efficiency, as is typically required in high-contrast microscopy applications, remains a challenge. Here we demonstrate curved GaAs metagratings integrated on vertical-cavity surface-emitting lasers (VCSELs) that enable on-chip illumination in total internal reflection and dark field microscopy. Based on an unconventional design that circumvents the aspect ratio dependent etching problems in monolithic integration, we demonstrate off-axis emission centred at 60° in air and 63° in glass with > 90% and > 70% relative deflection efficiency, respectively. The resulting laser beam is collimated out-of-plane but maintains Gaussian divergence in-plane, resulting in a long and narrow illumination area. We show that metagrating-integrated VCSELs of different kinds can be combined to enable rapid switching between dark-field and total internal reflection illumination. Our approach provides a versatile illumination solution for high-contrast imaging that is compatible with conventional microscopy setups and can be integrated with biophotonics devices, such as portable microscopy, NIR-II range bioimaging, and lab-on-a-chip devices.

## Abstract (web summary)

Integration of curved GaAs metagratings onto VCSELs enables a compact on-chip illumination solution for high-contrast microscopy, making it a versatile choice for light sources in lab-on-a-chip devices.

## Graphical abstract

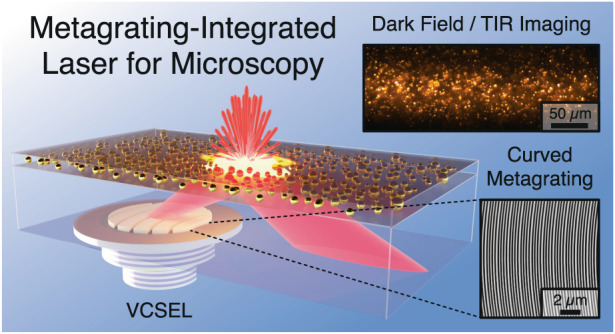

## Introduction

Wavefront engineering using collections of subwavelength nanostructures—or metasurfaces—started with simple demonstrations of lensing and anomalous refraction^1,2^ but has now evolved toward a variety of complex applications, including ranging^3^, polarization imaging^4^, holography^5^, optical manipulation^6^, and nonlinear photonics^7^. However, metaoptics are so far mostly used as stand-alone components, even though one of the most enticing advantages over traditional optics—fabrication process compatibility—intuitively suggests direct integration with optoelectronic devices. Indeed, recent reports demonstrated such integration on LED^8^, OLED displays^9^, and semiconductor lasers^10^. One application area that could significantly benefit from further progress in this direction is optical biosensing and microscopy, which still heavily rely on bulky external components such as lasers and compound microscopes. Flat metaoptics integrated with on-chip light sources, detectors, and microfluidics, could lead to extremely compact and cost-effective biophotonics devices able to drastically facilitate life-science research and applications.

**Fig. 1:**
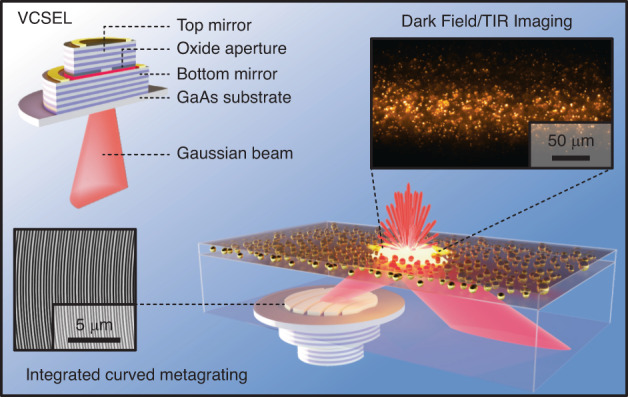
Overview of the metagrating-integrated laser for microscopy concept. Schematic illustration of an oxide-confined GaAs-VCSEL and its integration with a metagrating that produces off-axis emission at high angles for high-contrast DF and TIR microscopy

In recent years, the field of biophotonics has placed significant emphasis on label-free techniques for analyzing individual nanoscopic objects like biological nanoparticles, biomacromolecules, and drug carriers, primarily utilizing light scattering methods^11–15^. The scattering cross-section of sub-wavelength particles decreases rapidly with decreasing particle size, which necessitates efficient reduction of background caused by unscattered light. The traditional means of achieving this are various types of dark-field (DF) or total internal reflection (TIR) illumination setups based on ultrahigh numerical aperture (NA) immersion optics or waveguides coupled to bulky external light sources^16–18^. Full integration of a miniature light source into a planar chip for such high-contrast illumination has not yet been demonstrated, though a recent report on metasurface-coupled TIR microscopy based on an external light source indicates its feasibility^19^. In this work, we present curved metagratings monolithically integrated on vertical cavity surface-emitting lasers (VCSELs) with the aim to produce efficient emission at high deflection angles for high-contrast DF and TIR microscopy (Fig. 1).

The VCSEL is a semiconductor emitter that, because of its high efficiency and suitability for wafer-scale production, is widely used in face recognition, proximity sensors, augmented reality, among other applications. VCSELs are well-suited for monolithic integration with metasurfaces because they can be designed for single-mode emission normal to the epitaxial structure of the chip, which means that the beam shaping structure can be easily integrated in the process flow for fabrication of bottom-emitting VCSELs on transparent substrates. There have been several reports on structures etched into the VCSEL facet that modify its emission, for example into beam arrays^20^, orbital angular momentum beams^21^, and deflected circularly polarized beams^22^. Nanostructures have also been integrated with the mirrors of the VCSEL cavity to provide both optical feedback and to generate orbital angular momentum^23^ or circularly polarized output^24^.

The most widely used and the most general form of a beam shaping metastructure is a phase-gradient metasurface. It consists of discrete building blocks placed on the nodes of a uniform, subwavelength lattice by matching their calculated phase and amplitude response to the phase map of the intended functionality. Pioneering results on monolithic integration of such phase-gradient metaoptics and VCSELs was provided by Genevet and co-workers^25–27^. They showed on-chip beam shaping, including collimation, deflection, Bessel and OAM beam generation, etc. The phase mapping approach works well in the paraxial regime, where angles of deflection are small, but it fails at high deflection angles. In such high NA conditions, the spatial extent that encompasses a 2π linear phase gradient becomes comparable to the subwavelength unit cell size of the metasurface, leading to either insufficient or varying spatial sampling rate^28^. It is in principle possible to circumvent this problem by optimizing a full (2π phase space) super-unit cell^28,29^, but this approach generally leads to open areas of varying width between structures which, in the case of monolithically integrated metasurfaces, is problematic because of the effect known as aspect-ratio dependent etching (ARDE).

Metasurfaces are typically fabricated by a top-down process—etching the high-index material (e.g., silicon on a fused silica substrate) through a lithographically defined mask. The process of etching, especially at the nanoscale, is often limited by ARDE, which manifests as a reduction of etch rate as the ratio of the etched depth to the opening in the mask becomes larger. An etch-stop layer is the common solution, because it allows to prolong the etch time and let smaller openings to catch up. In this case it means depositing another material on the backside of the VCSEL chip with reasonable etching selectivity against GaAs. However, this would inevitably lead to a less efficient device and negate the advantages of monolithic fabrication on a pristine GaAs crystal because of defect induced absorption, refractive index and thermal expansion discontinuities.

Here we circumvent this problem by instead using metagratings, or subwavelength binary high-contrast gratings, which can be considered a proto-concept of metasurface flat optics^30–35^. The metagratings are optimized such that all etched trenches have equal width, which completely eliminates ARDE and results in >90% deflection efficiency. Furthermore, based on a simple axicon concept, we introduced grating curvature able to collimate the inherently divergent VCSEL emission out-of-plane while in-plane divergence is maintained. The fabricated structures are interfaced to microfluidic chips for proof-of-concept DF and TIR microscopy of nanoparticles. We also demonstrate switchable DF/TIR over a wide field-of-view using on-chip illumination.

## Results

### Concept of an offset axicon metagrating

The metasurface concept that we use in this paper is based on a uniform metagrating with constant period and unit cell size across the sample. Such a metagrating can be transformed into an axicon by curving the ridges into concentric circles. Axicons, like gratings, produce a constant deflection angle, but the angular orientation changes from unidirectional to conical. In terms of phase, axicons exhibit a linear phase gradient radially and a hyperbolic phase gradient tangentially. Consider an axicon that is radially offset with respect to the incident beam by distance *x*_*0*_. If the beam width is small in comparison to *x*_*0*_, it will experience the linear phase gradient defined by the NA of the axicon along the *x*-axis, but a hyperbolic phase gradient will be imprinted along the *y* axis. To approximately collimate the incident divergent VCSEL beam and simultaneously deflect it, we need to find the offset *x*_*0*_ such that the difference between this hyperbolic phase and the phase distribution of incident beam approaches zero. Thus

$${n}_{1}{NA}\sqrt{{{x}_{0}}^{2}+{y}^{2}}-{n}_{2}\left(\sqrt{{y}^{2}+{f}^{2}}-f\right)\to 0,$$

where *NA* = *sin* α is the numerical aperture of the axicon in air, *n*_*1*_, *n*_*2*_ are the refractive indices of air/glass and GaAs, respectively, and *f* is the negative focal length of a diverging spherical beam corresponding to the distance from the VCSEL aperture to the interface. The result will be an off-axis, quasi-collimated emission—approximately collimated out-of-plane but maintaining divergence in-plane (see Supplementary Information and Fig. S1 for a more detailed description).

### Design and fabrication of efficient metagratings

We start by optimizing a regular metagrating for a deflection angle that can be easily captured using a high-NA dry objective for characterization. We settled for 60° deflection in air, but this is not the limit. Following the classical diffraction equation *d* sin *θ* = *mλ*, this angle corresponds to a diffractive period *d* = 1127 nm for *λ* = 984 nm, which is the VCSEL emission wavelength. Usually, these kinds of metagratings follow either the filling factor design or the constant period design (see Fig. S2). The width of trenches in both cases vary, which leads to ARDE (Fig. S6). To eliminate ARDE, we focus on the trenches themselves in the design. We used finite element modelling and set up the simulation in terms of trench position within the diffractive unit cell and constrained them to be equal in width. This ensures that the eventually fabricated structure height is uniform across the whole metagrating.

**Fig. 2:**
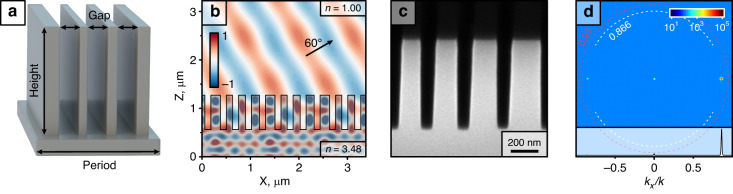
Design and fabrication of a metagrating in GaAs for efficient wide-angle deflection. **a** 3D render of the diffractive unit cell comprising the metagrating; **b** Calculated *H*_*y*_ field distribution of a metagrating in GaAs, deflecting normally incident plane wave to 60° in air with 75% total efficiency (96.1% relative to total transmittance); **c** SEM micrograph of the fabricated metagrating in GaAs; **d** Measured Fourier plane image (log_10_ intensity scale) and its cut at *k*_*y*_ = 0 (linear intensity scale). Dashed lines mark *k*_*x*_/*k* = 0.95 (maximum collection angle) and *k*_*x*_/*k* = 0.866 (target deflection angle)

We first performed simulation based on periodic boundary conditions and 3–5 trenches in GaAs (Fig. 2a) for *λ* = 984 nm light incident from the substrate side and polarized perpendicular to the grating lines. We achieved the highest efficiency using 4 trenches. The simulation converges to a solution with 75% total diffraction efficiency (T_+1_) and 96% relative efficiency (T_+1_/(T_0_ + T_+1_ + T_−1_)) (see Table S1 for geometric parameters and Fig. S3 for tolerance to fabrication errors). The resulting *H*_*y*_ field distribution is shown in Fig. 2b.

We fabricated the calculated structure in a 200 × 200 µm^2^ area on a GaAs substrate using a switching etching method that allows high aspect ratio trenches with nearly vertical sidewalls (see *Methods* for details). A SEM micrograph of the cross-section of a metagrating for deflection in air is shown in Fig. 2c. Note the constant gap widths, which ensure constant depth of trenches and high fidelity to simulation.

The fabricated samples were investigated using a custom-built microscope (Fig. S11). An external diode laser (*λ* = 976 nm) was loosely focused to cover ~80% of the central part of metagrating. The resulting Fourier plane image and a *k*_*y*_ = 0 cut is shown in Fig. 2d. Note that the image is plotted in log_10_ scale to aid the visibility of miniscule 0 and −1 orders. Remarkably, this structure, operating in air, showed >91% relative deflection efficiency, close to the simulated 96%. We attribute this achievement to high fidelity to the simulation because of uniform etch rate across the metagrating.

### Quasi-collimated oblique VCSEL emission

**Fig. 3:**
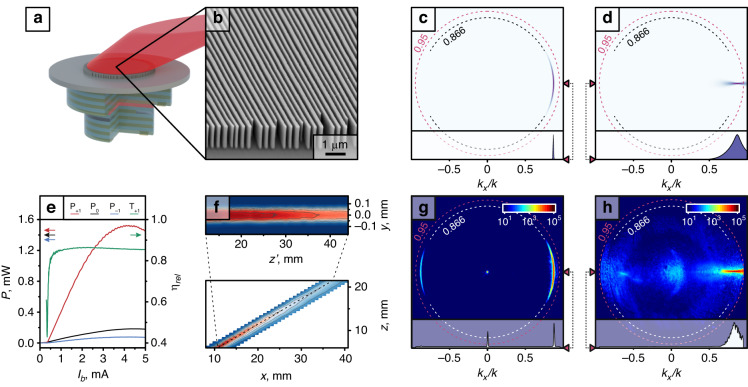
An integrated metagrating efficiently quasi-collimating the VCSEL emission and deflecting to 60° in air. **a** 3D render of the integrated metagrating concept; **b** SEM micrograph of the fabricated metagrating; **c**, **d** Simulated Fourier images of the curved metagrating illuminated by a plane wave (**c**) and a VCSEL (**d**) and their cuts at *k*_*y*_ = 0; **e** Free space measurement of the VCSEL power distributed into the separate diffraction orders and relative deflection efficiency of the integrated metagrating; **f** Beam profiles of the first diffraction order in the xz and z’y-plane; **g**, **h** Measured Fourier images (log_10_ intensity scale) of the curved metagrating illuminated by a plane wave (**g**) and a VCSEL (**h**) and their cuts at *k*_*y*_ = 0 (linear intensity scale); Dashed lines mark the *k*_*x*_/*k* = 0.95 (maximum collected angle) and *k*_*x*_/*k* = 0.866 (target deflection angle)

To address off-axis emission, we incorporated the metagratings described earlier onto VCSELs (Fig. 3a). VCSELs inherently exhibit significant divergence because of their small oxide aperture, leading to the inclusion of collimating lenses in commercial VCSELs. As discussed earlier, although a uniform metagrating design cannot emulate a regular lens, it can collimate along one axis by exploiting the offset axicon concept. Curved gratings are most often found as grating couplers in silicon photonics^36^ and serve a similar function.

We arranged the optimized metagrating structure in an axicon configuration by forming concentric rings. Following the design concept, we found the axicon offset *x*_*0*_ = 155 µm, which produces a hyperbolic phase distribution out-of-plane, closely matching the incident field phase resulting from propagation through GaAs (*n* = 3.48) from the VCSEL aperture at *f* = 625 µm. In the Fourier plane, an axicon produces a full circle with radius equal to the NA. If only an off-axis part of the axicon is illuminated, a corresponding section of the circle appears in the Fourier plane. This is illustrated in Fig. 3c where a calculated Fourier plane of a curved metagrating illuminated by an off-axis loosely focused beam is shown. The point that would describe a plane wave at an angle (such as in Fig. 2d) is modified by the metagrating such that it is displaced by a distance equal to the deflection angle but extended along a circle of the same radius. Therefore, a section of a circle with *k*_*x*_/*k* = 0.866 radius, corresponding to 60° deflection angle, can be seen.

Indeed, when a metagrating characterized by Fig. 3c is illuminated by a divergent VCSEL emission, the beam becomes collimated in the *k*_*y*_ direction and maintains divergence along *k*_*x*_ as illustrated in the calculated Fig. 3d. The emission that would be described by a Gaussian peak of radius corresponding to the divergence of the VCSEL is modified by a displacement defined by the deflection angle and the profile is changed to a line that retains the length defined by the initial Gaussian divergence. Therefore, the emitted beam emerges collimated out-of-plane but maintains divergence in-plane, forming a light sheet with a Gaussian intensity distribution.

Following this reasoning, we fabricated curved metagratings optimized to deflect light 60° in air (Fig. 3b) on a GaAs substrate and on the substrate of a VCSEL in a bottom-emitting configuration. First, we measured the latter in a free space setup and evaluated the power distributed among the diffraction orders (Fig. 3e). All fabricated devices demonstrated a relative deflection efficiency of >80% as long as the VCSEL had a stable polarization state (see Supplementary Material for a full VCSEL characterization). Since the deflected light in the diffraction orders diverges in-plane it is possible that we did not pick up all power, meaning we likely underestimated the efficiency. Because of the very high transmission, the ripple seen in the output power in the IPV curve, which is due to interference caused by the metasurface back-reflection, is very small. We have also imaged the first diffraction order beam profile in free space 6–22 mm away from the chip. In Fig. 3f we show the xz and z’y cuts of the beam profile (z’ follows the diffracted beam). The beam is clearly deflected 60° from the normal and has a very small divergence out-of-plane (along the y axis) but diverges in-plane (along the x-axis), which confirms the quasi-collimation caused by the curvature of the integrated metagrating (see Supplementary Information Fig. S14 for the measurement setup).

We evaluated both the integrated metagrating and its stand-alone version in the microscopy setup. The efficiency estimation of the latter showed a similar result to the grating without curvature, >93%. Fig. 3g shows the measured Fourier plane of this metagrating, demonstrating excellent agreement with the simulation result in Fig. 3c (note that the diffracted orders in the *k*_*y*_ = 0 cut are spread out in the azimuthal direction, causing the central 0:th order to appear very strong). The measured Fourier plane of the integrated metagrating is shown in Fig. 3h. The efficiency is also very high, estimated to >86% (for efficiency calculation details see Supplementary Material), matching the free space measurement. The emission profile acquires the linear shape offset to the designed deflection angle as predicted in Fig. 3d. The results thus demonstrate that these integrated devices produce highly efficient quasi-collimated off-axis emission.

### VCSELs with integrated metagratings for on-chip microscopy illumination

Traditionally, TIR illumination is achieved using bulky prisms or expensive high NA objectives that limit the field of view. Waveguide-based excitation of evanescent fields solves some of these issues but still requires external excitation and light coupling, usually provided from the side of the waveguide chip^16–18^. A recent report showed a metasurface-coupled TIR microscopy chip but it used an external light source nevertheless^19^. We therefore explored the option to use the metagrating integrated VCSELs for TIR and DF microscopy applications. We used analogous structure as described above and optimized it for glass (*n* = 1.51) for a deflection angle of ~63°, which is just above the critical angle for a glass/water interface. This is not the upper angular limit, but it gives larger fabrication tolerances while still fulfilling the desired application objective. With the new unit cell size of 730 nm and re-optimized parameters, listed in Table S2, we obtained 70% total and 83% relative diffraction efficiency. To ensure a reasonable fabrication complexity, we limited the minimum ridge and gap widths to 100 nm and 75 nm, respectively (see Fig. S4 for tolerance to fabrication errors). We found that the optimal number of gaps per unit cell was 3; 4 could not fit into the unit cell considering the fabrication constraints and 2 did not produce higher efficiency. A fabricated stand-alone metagrating of this design showed high relative efficiency of >74% when illuminated using an external laser.

**Fig. 4:**
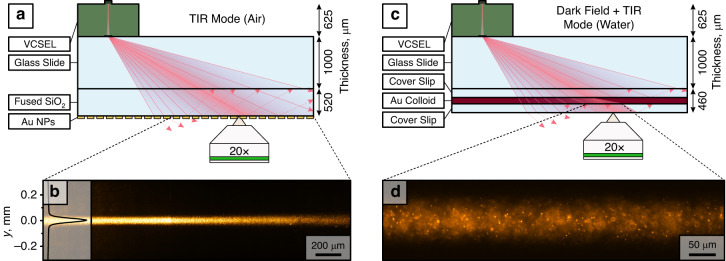
DF and TIR microscopy using on-chip VCSELs with integrated metagratings. **a** A schematic of the on-chip microscopy setup, where Au nanoparticles on a fused silica substrate are illuminated by TIR laser light; **b** Stitched optical micrographs of light scattered by Au nanoparticles fabricated on a glass/air interface, illuminated by TIR VCSEL light; **c** A schematic of the on-chip microscopy setup, where Au nanoparticles in a colloid solution are illuminated by DF-TIR laser light; **d** An optical micrograph of light scattered by colloidal Au nanorods (180 × 88 nm^2^) illuminated by DF-TIR illumination

The metagratings were monolithically integrated on VCSELs as before and then glued on a standard 1 mm thick microscopy slide using an optical adhesive (Norland NOA60). The resulting illumination module was first tested in a proof-of-principle demonstration of TIR microscopy in air (Fig. 4a). The module was interfaced to a fused silica substrate using immersion oil. The substrate contained a dense array of scatterers (Au nanoparticles, 200 × 200 nm^2^, 400 nm periodicity) fabricated by electron-beam lithography, which allowed the illuminated area to be seen and quantified by imaging through a 20×, NA = 0.5, dry objective, as shown in Fig. 4b. The illumination area has a Gaussian intensity profile along *x* and *y*, but the extension along *x* is much larger because of the maintained divergence this direction, as described above. The result is an almost uniform illumination along *x* (~15% variation from side to side over 650 µm field of view), whereas the intensity variation along *y* is much narrower and clearly Gaussian (FWHM ≈ 40 µm).

As a second demonstration, we interfaced the illumination module to a microfluidic cell containing a colloidal solution of Au nanorods (Fig. 4c). The range of illumination angles is centred on 63° but spans 30°–90°. The colloid is therefore illuminated in DF within the area covering the angular range below the critical angle, and in TIR within the area above that range. Unfortunately, the TIR area is now slightly contaminated by DF illumination reflected from the air-glass interface of the microfluidic cell, resulting in mixed TIR/DF conditions and background from particles diffusing in the liquid volume beyond the evanescent field region (Fig. 4d).

**Fig. 5:**
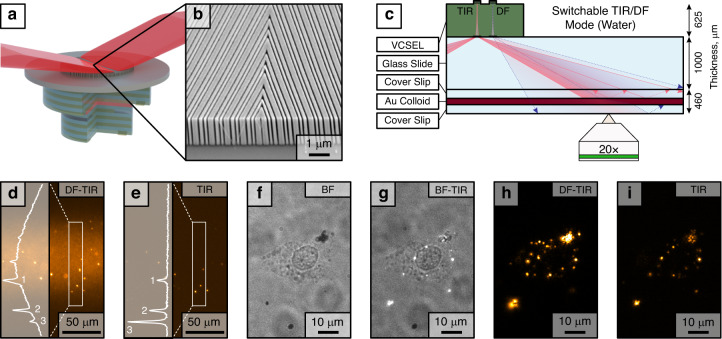
VCSEL with an integrated metagrating in a mirror-symmetric configuration provides clean TIR illumination. **a** 3D render of the integrated mirrored metagrating concept; **b** SEM micrograph of the fabricated metagrating; **c** Schematic of the on-chip microscopy setup, where Au nanoparticles in a colloid solution are illuminated by only TIR laser light; **d**, **e** Optical micrographs of light scattered by colloidal Au nanorods (180 × 88 nm^2^) by combined DF/TIR illumination (**d**) and TIR illumination only (**e**), insets show corresponding intensity cuts; **f**–**i** Optical micrographs (60× objective) of a sample with nanorod-incubated cells: bright field (**f**), bright field with TIR (**g**), DF-TIR (**h**), and TIR only (**i**) modes

To solve the issue above, we developed a switchable DF/TIR illumination module as the final demonstration (Fig. 5). This could be accomplished by letting the emission from two adjacent but independent VCSELs overlap the same observation area. The metagrating interfaced to the first VCSEL is identical to the case above, that is, it produces mixed TIR/DF illumination as in Fig. 4d. The metagrating interfaced to the second VCSEL, however, is instead divided in two mirror-symmetric halves that each emit at angles in the range starting from 63° (Fig. 5a and b). The two resulting counterpropagating beams thus both provide TIR, but only one is used to illuminate the sample while the other is efficiently directed away from observation area (Fig. 5c). A calculated Fourier image is shown in Fig. S5.

To demonstrate the on-chip illumination switching we used the same colloidal Au particles as above and focused on the top interface of the cell. By switching between the two adjacent VCSELs we took successive pairs of images, one in which the observation area was exposed to mixed TIR/DF illumination (Fig. 5d) and the other only to TIR (Fig. 5e). It is obvious that the former contains strong background from diffusing out-of-focus particles illuminated in DF, whereas the latter only shows particles adsorbed or very close to the interface against a very weak and flat background. Finally, we tested the device on a sample with live human endothelial HMEC-1 cells (see Supplementary Information for details) that had been incubated with Au nanorods (Fig. 5f–i). Since the laser chip does not obscure transmitted light, bright field (BF) images could also be taken and show a cell with weak contrast. DF image captured using the illumination module highlights the nanoparticles within the cell. Switching on the adjacent VCSEL in the TIR mode allowed to highlight only the nanoparticles that are close to the glass surface. BF-TIR combined mode is also available by using our module with standard BF illumination.

## Discussion

In summary, we have proposed a highly efficient metagrating structure uniquely designed to accommodate monolithic integration on a VCSEL substrate and to achieve very high >86% relative deflection efficiency. We achieved high fidelity between calculated design and fabrication by designing the metagrating with constant trench widths between individual nanostructures and effectively eliminated any ARDE effect that deteriorates performance. The relative deflection efficiency achieved comes close to or even surpasses the results achieved using more complicated fabrication procedures, such as multi-level lithography^37^. Furthermore, we demonstrated a novel axicon design that provides high angle deflection and simultaneous quasi-collimation via the curvature of the metagrating. The design relies on offsetting the metagrating-based axicon by a distance such that the tangential hyperbolic phase gradient matches the input phase generated by the VCSEL.

We applied the metagrating-integrated VCSELs as illumination sources for high-contrast microscopy. These proof-of-principle results demonstrate high quality switchable DF and TIR illumination capabilities, with the main advantages compared to state-of-the-art illumination devices being that the light source is cheap, efficient, very compact and comes with a built-in and pre-aligned light-coupling solution. Since the illumination module does not obscure the microscope field-of-view, it can be easily combined with conventional bright-field or fluorescence observation. Moreover, the device could be integrated with microfluidic flow chips for in-situ observation, further approaching full lab-on-a-chip integration^38^. One notable benefit in this regard would be the ability to utilize the elongated emission from one or multiple VCSELs to illuminate targeted microfluidic channels. This would effectively decrease the background noise caused by scattered light from the chip itself.

A major advantage of light sources based on VCSELs is that several emitters on the same chip can be configured to produce different illuminations, which allows for quick switching during a measurement, as in our demonstration of TIR-DF switching. Combined with a variation of curvature in the metagratings to change the illuminated area size, the location of the area of interest can be facilitated before switching to a more concentrated illumination with full collimation. Such integrated multi-functional light source can become a versatile platform for rapid screening of samples without the need of full microscope reconfiguration, e.g., switching between TIR and DF condensers.

Ideally, our proposed illumination module would be combined with fluorescence imaging, but there is so-far a lack of efficient fluorophores operating at ~1000 nm wavelength. However, this is an actively researched area because excitation within the NIR-II range (1000–2000 nm) features dramatically reduced auto-fluorescence and light toxicity compared to conventional visible-range excitation. We therefore expect our demonstration to prove useful also for fluorescence imaging, once suitable functional fluorophores are readily available^39–41^.

Finally, the metagrating concept demonstrated here can easily be reoptimized and integrated with both, InP-based VCSELs^42^ that emit at longer wavelengths, as well as with VCSELs emitting in the visible wavelength spectrum, though these are still at an early development stage^43,44^.

## Materials and methods

### Grating simulations

Metagratings were simulated using the Wave Optics module of COMSOL Multiphysics software in 2D. Periodic boundary conditions were defined on the sides of the computation domain, while the bottom and top boundaries were assigned as exciting and receiving ports with in-plane polarization. To find the optimal structure, the optimization module was used with the Nelder-Mead algorithm and the transmittance of +1 diffraction order as the figure of merit. Depending on the unit cell size, either three or four trenches were defined in the calculation domain and were allowed to change their position within the unit cell freely, as well as their width and height. The trenches were constrained to be equal width. The optimization was allowed to run until converged. GaAs refractive index data was taken from Papatryfonos et al^45^.

### VCSEL fabrication

The VCSEL epitaxial structure is grown by MOCVD and features an active region with three strained InGaAs quantum wells sandwiched between two distributed Bragg reflectors (DBR), with 28 and 20 AlAs/GaAs and/or AlGaAs/GaAs pairs on the topside and substrate side, respectively, for bottom emission through the GaAs substrate. The top DBR has one AlGaAs layer with a 98% aluminium content which is selectively oxidized to form an oxide aperture for lateral current and optical mode confinement. The VCSELs were fabricated using standard processing steps of oxide confined GaAs-based VCSELs. First, Ti/Pt/Au top contact rings were evaporated on a highly doped p-type layer on the top DBR. After which alignment marks are transferred to the backside of the chip to align the metagrating with the centre of the VCSEL. A VCSEL mesa with 21 µm diameter is then dry etched by an inductively coupled plasma (ICP) reactive ion etching (RIE) using Ar/SiCl_4_ gas mixture. The oxide aperture is created by selective wet oxidization at 420 °C. The targeted aperture size is 2 µm to achieve single-mode lasing. After oxidation, a Ge/Ni/Au bottom contact is evaporated and annealed to create an ohmic contact to the n-doped bottom mirror. The VCSEL chip is passivated by a 350 nm thick layer of SiN_x_ which is dry etched to open connections to large, sputtered Ti/Au pads for electrical injection. Finally, 250 nm of SiN_x_ is sputtered to protect the VCSELs during the following metasurface fabrication. More details can be found in the supplementary information.

### Metasurface fabrication

Either semi-insulating GaAs substrates or VCSEL chips in the bottom-emitting configuration were used to fabricate monolithic metagratings. SiO_2_ layer was sputtered on the substrate to serve as an etching mask. Optimized patterns were exposed in positive ArP 6200.13 e-beam resist. After development, Ni was evaporated and lifted-off. Patterns were then etched into SiO_2_ in CF_4_/O_2_ plasma. Finally, GaAs was etched using a switching process, alternating between SiCl_4_ and O_2_ plasma processing steps. Since there is no etch-stop layer, the etching depth was controlled by timing and stopping the process manually. More details can be found in the supplementary information.

### Optical setup

The fabricated metasurfaces on GaAs substrates and integrated on VCSEL chips were characterized using a custom-built optical microscope equipped with 40× NA = 0.95 dry objective. GaAs substrates were illuminated using a loosely focused, linearly polarized 976 nm laser beam. A lens focused on the back-focal plane of the objective could be inserted for Fourier plane imaging. The relative efficiency of the metagratings was determined by capturing Fourier images, subtracting the background, integrating the pixel values in the corresponding areas of the Fourier plane, and referencing to the total sum of pixel values on the image. DF and TIR images of nanoparticles and cells were taken in the same setup using a 20×, NA = 0.5 and 60×, NA = 0.7 dry objectives respectively.

### Free space measurements

The power in each diffraction order was captured using large-area silicon photodiode, Hamamatsu S2281-01. Three IPV measurements were performed for each device, one for each diffraction order of the VCSEL with the metagrating, by placing the photodiode at the appropriate angle for each diffraction order. The beam profiles were measured by illuminating a diffusive plate, Ophir WB-I SWIR. The image on the diffusive plate was then focused and captured by a CCD camera, Ophir SP932U.

### Supplementary information

Supplemental Information

## Data Availability

The data supporting the findings of this study are available from the corresponding author on reasonable request.
