# Supplementary material for: High-angle deflection of metagrating-integrated laser emission for high-contrast microscopy
Source: Light Sci Appl. 2023 Oct 13;12:251. doi: 10.1038/s41377-023-01286-0 (PMC10576095; doi:10.1038/s41377-023-01286-0)
Supplement: Supplementary file 1 — Supplemental Information [file 41377_2023_1286_MOESM1_ESM.docx]

High-Angle Deflection of Metagrating-Integrated Laser Emission for High-Contrast Microscopy

Mindaugas Juodėnas^1*^, Erik Strandberg^2^, Alexander Grabowski^2^, Johan Gustavsson^2^,
Hana Šípová-Jungová^1^, Anders Larsson^2^, and Mikael Käll^1*^

^1^Department of Physics, Chalmers University of Technology, 412 96 Gothenburg, Sweden.

^2^Department of Microtechnology and Nanoscience, Chalmers University of Technology, 412 96 Gothenburg, Sweden.

^*^Corresponding authors: mindaugas.juodenas@chalmers.se; mikael.kall@chalmers.se

# Supplementary Information

## Axicon metagrating concept details

Axicons imprint the incident light with a phase delay of the form:

$\phi_{axicon}(x,y)=-\frac{2\pi n_{1}}{\lambda}NA\sqrt{{(x-x_{0})}^{2}+{(y-y_{0})}^{2}}$,

where *n_1_* is the refractive index of the medium (air/glass), *λ* is vacuum wavelength, *NA* = *sin* α is the numerical aperture in air, and (*x_0_*, *y_0_*) is the offset between the optical axis of the axicon and the reference coordinate system. This equation defines a cone. Consider the intersection of this cone with a plane *x* = 0. If the axicon is centred on the (0, 0) coordinate, the plane *x* = 0 coincides with the axis of the cone and the intersection is a line with an abrupt trend change at the apex. This is no good for any collimation, because the phase of incident light is not linear, but has the spherical form:

$\phi_{inc}(x,y)=-\frac{2\pi n_{2}}{\lambda}(\sqrt{x^{2}+y^{2}+f^{2}}-f)$,

where *n_2_* – refractive index of the medium (GaAs), *λ* – wavelength in vacuum, *f* – the negative focal length of a diverging spherical beam corresponding to the distance from the VCSEL aperture to the interface. However, if we displace the axicon by a distance *x_0_*, as illustrated in **Figure S1**, the intersection with the cone is now a hyperbola. Importantly, the orthogonal plane *y* = *0,* will still intersect the cone with a line. Following this, one needs to find the offset *x_0_* so that the difference between the hyperbolic phase of the offset axicon and the phase distribution of incident light along the *y* axis approaches zero:

$$\Delta\phi\left( x=0,y \right)= \phi_{axicon}\left( y \right)- \phi_{inc}\left( y \right)\to0$$

$n_{1}NA\sqrt{{x_{0}}^{2}+y^{2}}-n_{2}\left( \sqrt{y^{2}+f^{2}}-f \right)\to0$.


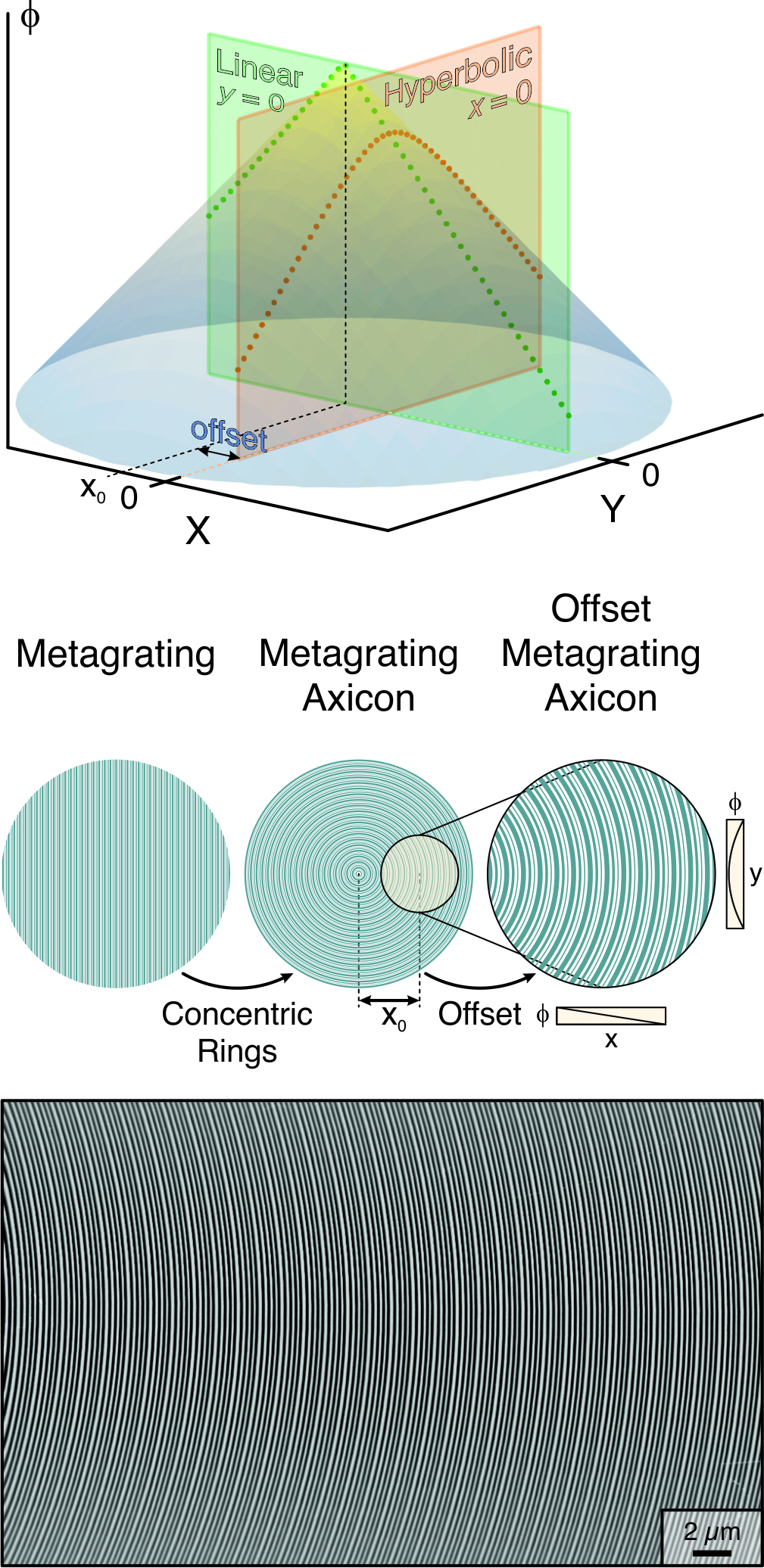


**Figure S1.** Intersection of a conical phase profile and a plane parallel to its axis produces a hyperbola – an illustration of the offset axicon’s phase gradient effect on two orthogonal axes. A metagrating is transformed into an axicon by forming concentric circles and an offset subsection of it is fabricated (SEM micrograph).

To generalize, the phase gradient of an axicon offset by a distance *x_0_* will be approximately linear along the *x* axis and hyperbolic along the *y* axis. We say approximately because the phase gradient along the *x* axis when *y* ≠ 0 is also hyperbolic, but at sufficiently high offset *x_0_* it approaches a line.

This approximation starts to fail as the area of the metagrating becomes larger, because the parameters of the hyperbola depend on *x_,_* and we only optimize it for the central cut of the incident light at *x* = 0. However, as we show experimentally, it still works very well for small area metagratings, where the variation of the hyperbola is not significant.

The large NA of the axicon defining the deflection angle is what allows to exploit the curvature to quasi-collimate the Gaussian illumination. In fact, this is only possible in combination with large deflection angles. The design relies on offsetting the metagrating-based axicon by a distance such that the tangential hyperbolic phase gradient matches the input phase. This of course depends on the input light source, but in this paper, low-NA axicons will only produce a sufficient hyperbolic phase profile very close to the centre of the axicon. This causes either inclusion of the centre of the axicon in the metasurface, or a much smaller metasurface size; on top of that, the closer to the centre, the higher the variation of the hyperbolic phase profile along the radial direction, which causes the emission to adopt a bowtie-like shape in the far field.

## Metasurface design and simulation details

Metagratings, or blazed binary gratings, usually follow the filling factor design, where the diffractive period is subdivided into subunit cells that are filled by ridges of varying widths, or the constant period design, where these ridges with varying widths are placed in defined positions in the diffractive period. In this paper, we use the unconventional design of constant trenches to aid in the fabrication.


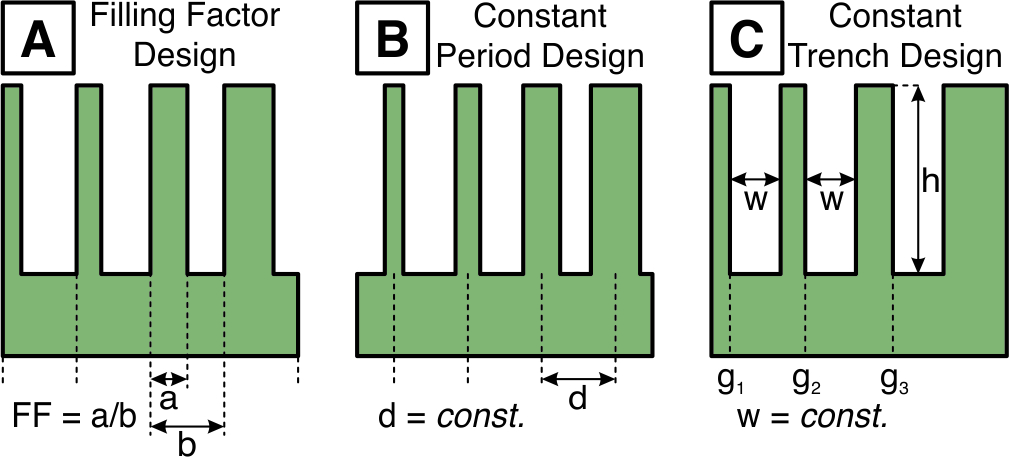


**Figure S2.** Comparison of different metagrating design approaches: A) the filling factor design; B) the constant period design; C) the constant trench design (this paper)

**Table S1.** Optimized parameters of metagrating in GaAs, deflecting 976 nm light to 60° in air with 75% efficiency.

|  | Position *g*, nm | Width *w*, nm | Height *h*, nm |
| --- | --- | --- | --- |
| Gap 1 | 59 | 125 | 710 |
| Gap 2 | 379 |  |  |
| Gap 3 | 629 |  |  |
| Gap 4 | 897 |  |  |


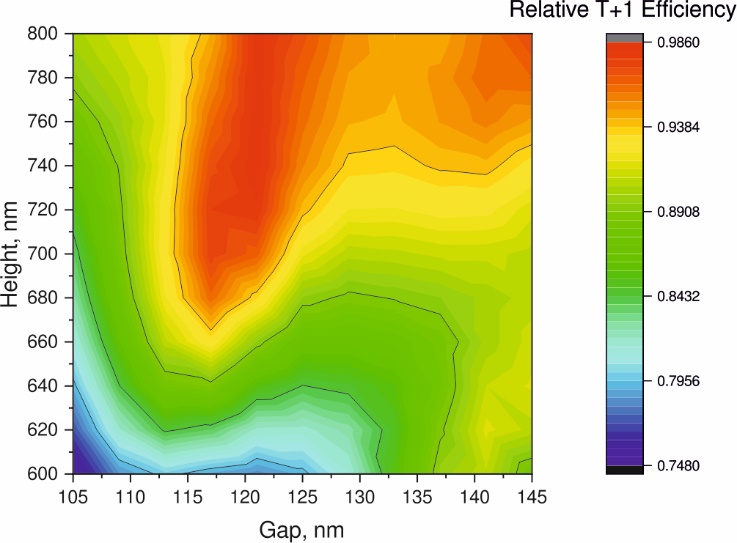


**Figure S3.** The relative deflection efficiency of a metagrating bending light to 60° in air. A sweep of trench width and height parameters.

**Table S2.** Optimized parameters of metagrating in GaAs, deflecting 976 nm light to 63° in immersion oil with 73% efficiency.

|  | Position *g*, nm | Width *w*, nm | Height *h*, nm |
| --- | --- | --- | --- |
| Gap 1 | 50 | 75 | 717 |
| Gap 2 | 278 |  |  |
| Gap 3 | 605 |  |  |


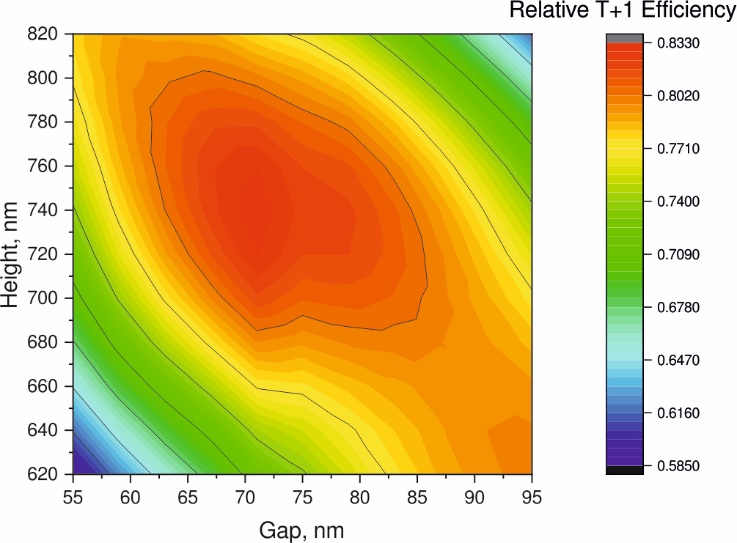


**Figure S4.** The relative deflection efficiency of a metagrating bending light to 63° in glass. A sweep of trench width and height parameters.


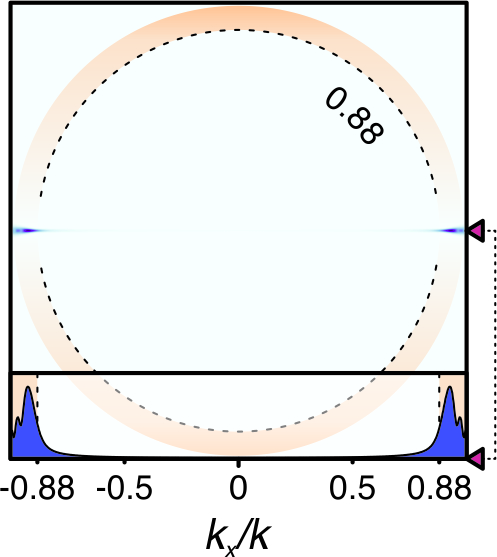


**Figure S5.** Simulated Fourier image of the curved metagrating illuminated by a VCSEL and its cut at *k_y_* = 0; Dashed lines mark *k_x_*/*k* = 0.88 (critical angle for glass/water interface), yellow shaded area indicates the TIR region.

## GaAs etching details

The ICP-RIE etching process consists of a looped sequence, where an etching step (SiCl_4_/Ar 8/42 sccm, 25 s) is exchanged with a passivating step (O_2_/Ar 2/48 sccm, 5 s) with intermediate purging steps (Ar 50 sccm, 5 s). 30W platen power, 25W ICP power, and 1.7 mT pressure are maintained throughout the process. The isolated passivation step forms SiO_2_ on the sidewalls only because horizontal surfaces are consistently cleaned by ion bombardment. The aspect ratio dependant etch rate is displayed in the inset of Figure S6.


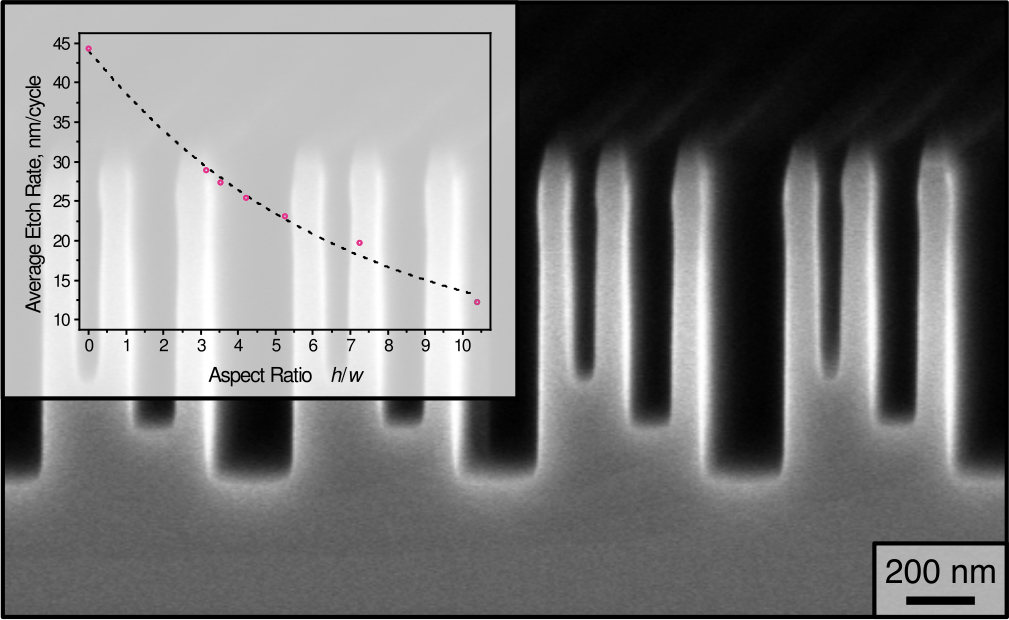


**Figure S6.** An SEM image of a metagrating illustrating aspect ratio dependent etching. Inset shows average etch rate data collected using the described process.

## VCSEL design details

In **Figure S7** we present a schematic of the fabricated bottom emitting VCSEL with an integrated metagrating, emitting at ~980 nm. Starting from the top, the VCSEL consists of an evaporated Au/Pt/Ti ring-shaped p-contact for electrical injection. The contact is deposited on a highly doped p-type GaAs-layer. The top p-type distributed Bragg reflector (DBR) consists of 28 pairs of GaAs/Al_0.90_Ga_0.10_As with graded composition interfaces and modulation doping for low electrical resistance. In the last mirror pair, close to the active region, one layer with higher aluminium content (Al_0.98_Ga_0.02_As) is inserted to allow for the formation of an oxide aperture by selective wet oxidation after etching a 21 µm diameter mesa through the top-DBR.


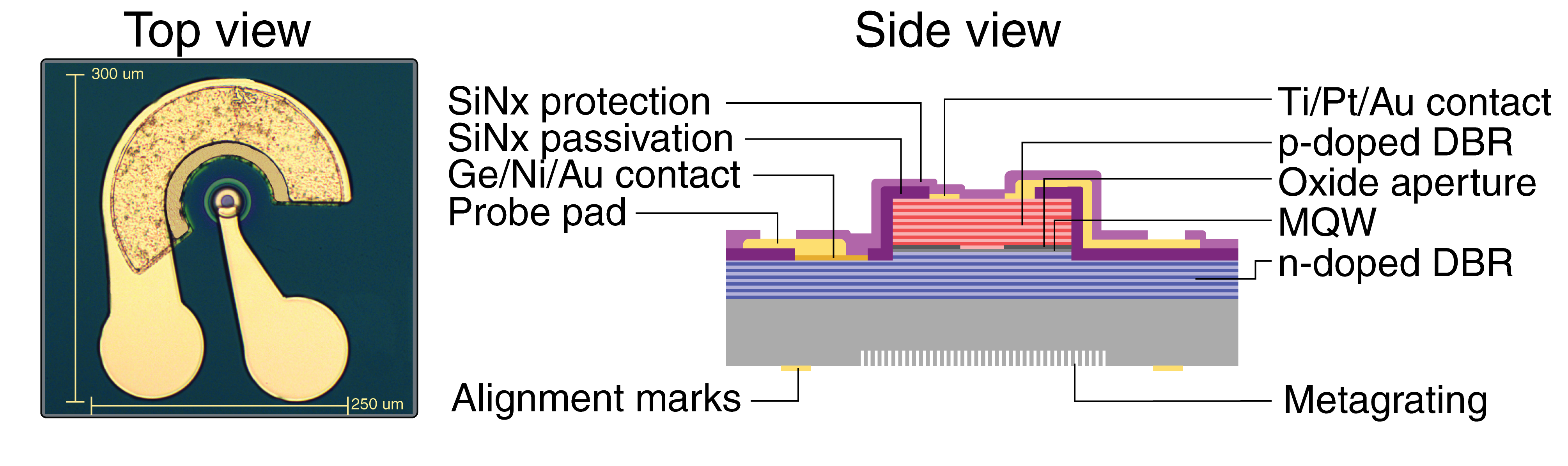


**Figure S7.** In the left figure is a microscope image of the finished metagrating VCSEL. In the right figure is a schematic view of the structure of the fabricate metagrating VCSEL. The full structure is described above.

The oxide aperture confines the current and the optical modes in the lateral direction. The oxide is electrically insulating, which creates a path for current through the aperture. It also has a low refractive index which creates a waveguide for optical confinement. With a small enough aperture, single-mode emission can be achieved. The oxide aperture is placed at a node of the optical standing wave and therefore the guiding is weak. This allows for the largest possible oxide aperture, and therefore the lowest electrical resistance, in single-mode operation.

The gain medium is located directly below the oxide aperture and consists of a 1λ thick separate confinement heterostructure with three 5 nm thick In_0.22_Ga_0.78_As QWs separated by barriers of GaAs_0.85_P_0.15_. The bottom n-type DBR consists of 2 pairs of GaAs/Al_0.90_Ga_0.10_As + 18 pairs of AlAs/GaAs with graded composition interfaces and modulation doping for low electrical resistance. A Ni/Ge/Au n-contact is deposited on the third DBR mirror pair layer below the active region and annealed for low contact resistance. The bottom emitting VCSEL structure is grown on a GaAs substrate. The substrate is undoped to avoid optical loss from free carrier absorption. The substrate is 600 µm thick to allow for the VCSEL beam to diverge before being incident on the metagrating. With a 2 µm oxide aperture the beam has a diameter (1/$e^{2}$) of 82 µm at the GaAs-air interface. A metagrating with a diameter of 200 µm will then catch 99% of the light emitted from the VCSEL.

The VCSEL has two layers of SiN_x_ for protection. The first SiN_x_ layer is 350 nm thick and passivates the crystal surface to only allow for current to flow through the deposited contacts. The second SiN_x_ layer is 250 nm thick and protects the VCSEL during metagrating fabrication.

## VCSEL Characterization

VCSELs without a metasurface were tested to verify their basic performance.

*Current-power-voltage (IPV) measurements.* The VCSELs are probed and biased with current from below threshold to beyond maximum output power at thermal rollover. The output optical power (P), bias current (I) and corresponding voltage (V) are measured. The IPV characteristics of the 2 µm oxide aperture VCSEL at room temperature can be seen in **Figure S8**. It has a threshold current of 0.3 mA and rolls over at 6 mA, with a maximum output power of around 2.8 mW. During the lasing phase, the linear increase in output power has a slope efficiency of 0.6 W/A.


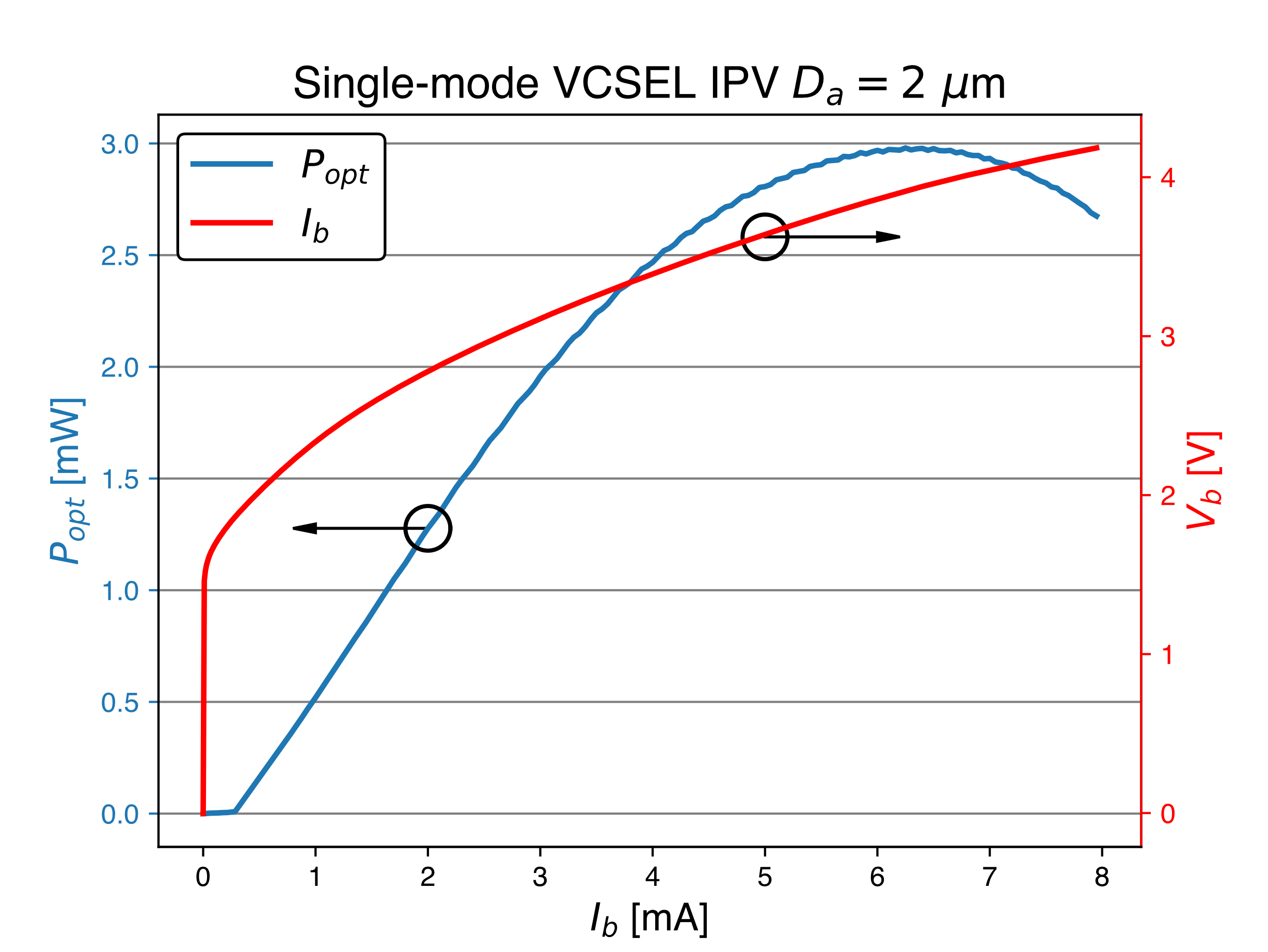


**Figure S8.** A representative IPV measurement from a VCSEL without an integrated metagrating. The laser starts to lase at 0.3 mA, rolls over at 6 mA with a maximum output power of 2.8 mW. The emitted beam needs to propagate through a GaAs-air interfaces at the bottom of the substrate. To reduce the reflections a SiNx AR coating was sputtered on the substrate side. The ripple that can be seen in the output power comes from interference with the reflection.

*Polarization current-power-voltage (PIPV) measurements.* The fabricated VCSELs do not have anything pinning the polarization. Since the VCSEL cavity is rotationally symmetric with respect to the substrate plane, the cavity modes are two-fold degenerate with orthogonal polarization. Therefore, in a single transverse mode VCSEL (without polarization pinning structure) the emitted beam can come from any of the polarization modes. The electric fields of the two degenerate modes, for a VCSEL grown on a $(100)$ oriented substrate, are usually oriented along the $[011]$ and $[0\bar{1}1]$ crystal axes. With no anisotropy, both modes have the same eigenfrequency and would therefore lase with the same intensity. However, in real VCSELs, due to weak anisotropies, the two polarization modes are generally separated. This leads to the polarization state of the VCSEL being unpredictable without a polarization pinning structure. Our VCSELs have an unpredictable polarization state, but the polarization mode that is oriented along the$[0\bar{1}1]$ crystal axis lases preferentially at low currents.

To characterize the polarization state of the VCSEL we performed the same IPV measurements, but with a polarizer inserted between the laser and the detector. Two IPV measurements were performed for every VCSEL, one with the polarizer blocking the electric field from the lasing mode oriented along the $[011]$ axis, the second with the polarizer rotated by 90$^{\circ}$, blocking the electric field along the orthogonal axis, $[0\bar{1}1]$. In **Figure S9** the general characteristics of the polarization resolved IPV measurement of the VCSEL can be seen. At low currents, the VCSEL has a preferential polarization state, $P_{opt, [0\bar{1}1]}$. At currents higher than 3 mA, both polarization modes are lasing. At 3 mA, the orthogonal polarization mode, $P_{opt, [011]}$, emits 1% of the total optical output power. We therefore have up to 2 mW of output optical power with predictable polarization, which is more than needed to demonstrate the dark-field and TIR microscopy functionalities. All measurements performed to characterize the deflected beam with the metasurface was performed at 1.4 mA, where the orthogonal polarization mode is strongly suppressed.

*Spectral measurements.* To measure the VCSEL spectrum, the emitted beam was focused onto the tip of an optical fibre and fed to an optical spectrum analyser. The spectrum for a 2 µm aperture VCSEL can be seen in **Figure S10**. A side-mode suppression ratio (SMSR) >30 dB was achieved at currents from just above threshold at 1 mA to beyond rollover at 7 mA. The resolution in the spectral measurements is not sufficient to resolve the nearly degenerate polarization modes. Therefore, only one peak appears in the spectra.


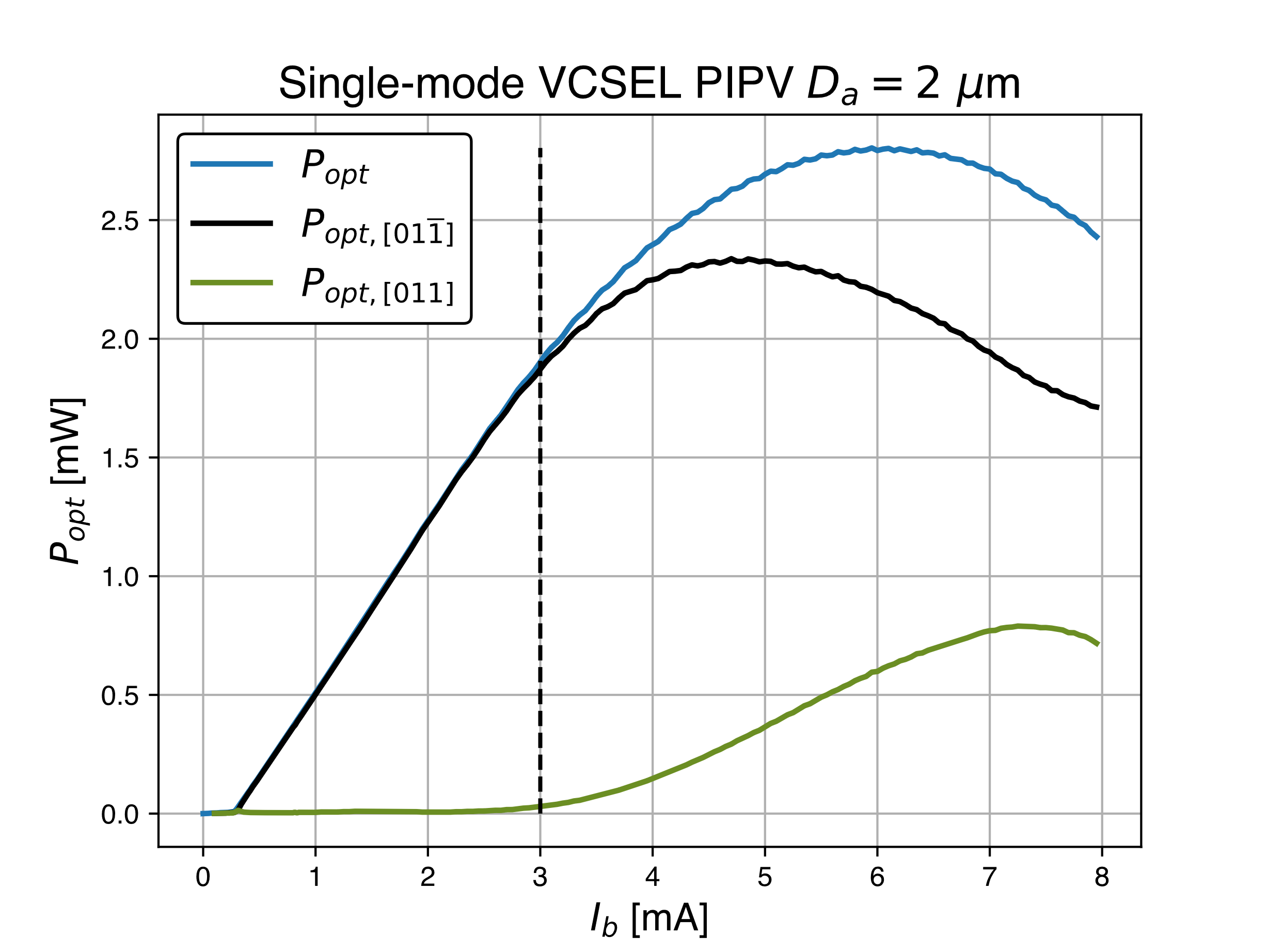


**Figure S9.** A representative PIPV of the same VCSEL. The VCSEL has a well-defined polarization state until ~3 mA. $P_{opt}$ is the total optical output power measured without the polarizer. $P_{opt, [011]}$ and $P_{opt, [0\bar{1}1]}$is optical output power associated with the respective crystal axes.


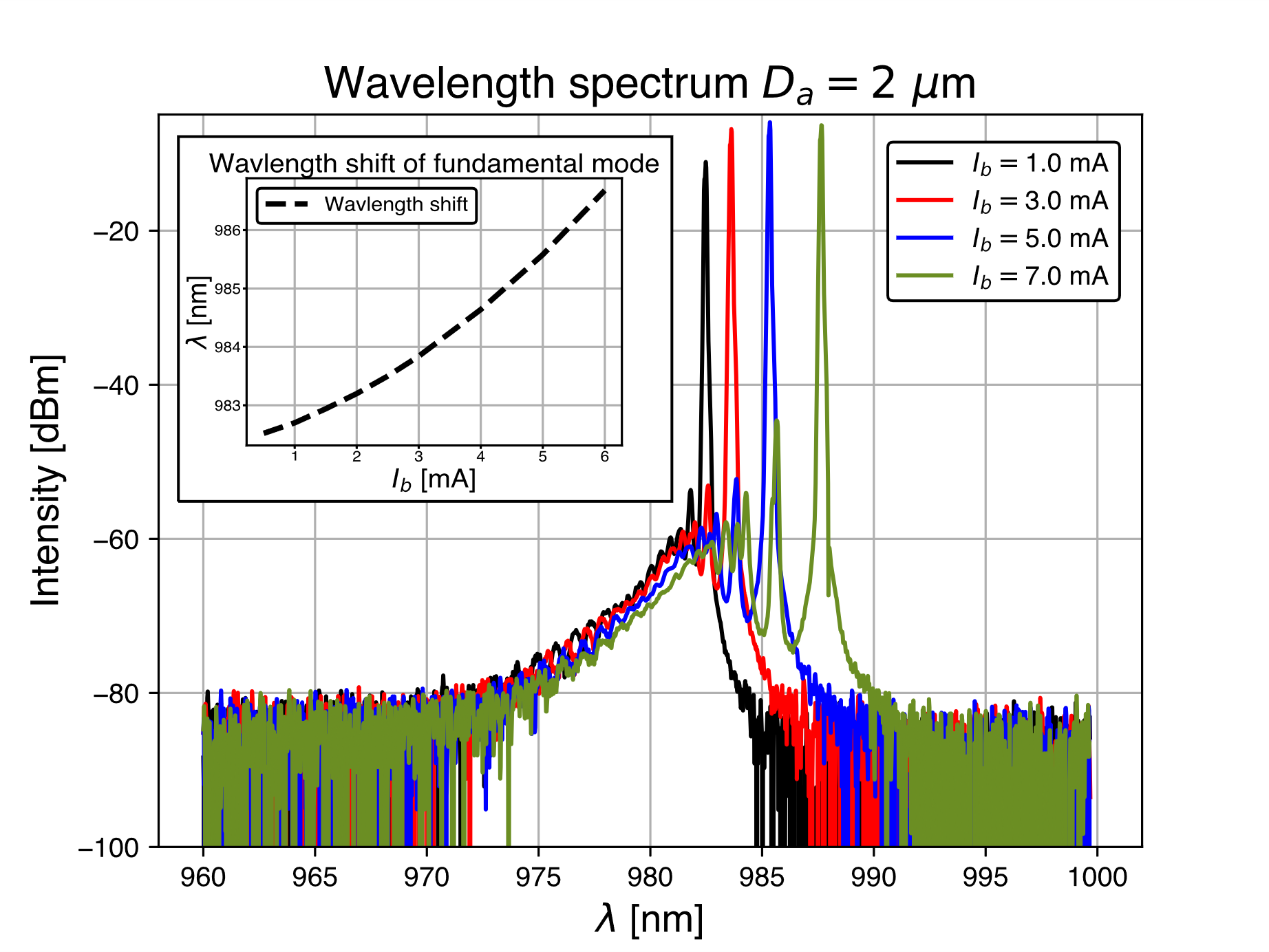

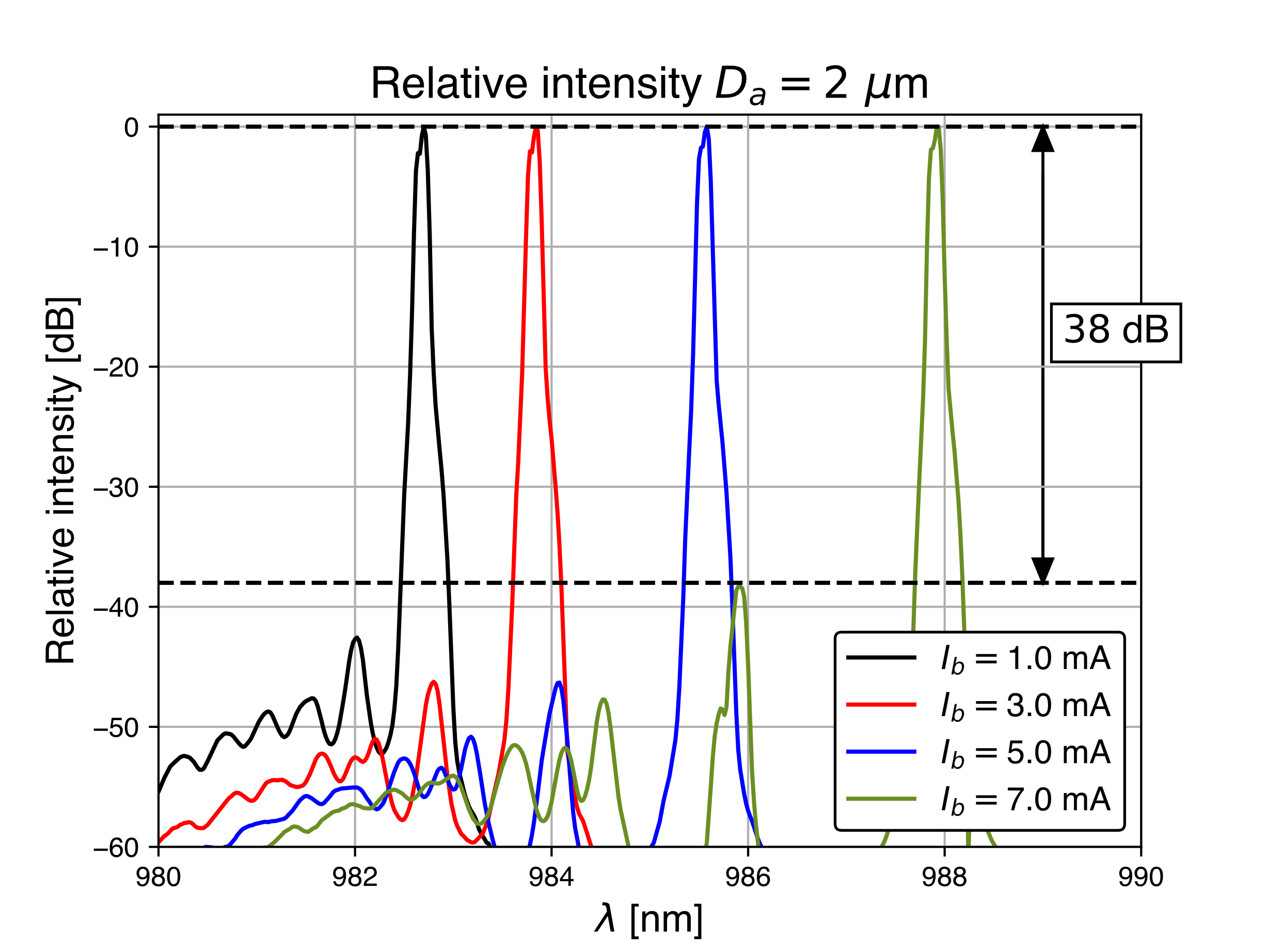


**Figure S10.** Spectra of the 2 µm aperture VCSEL. The inset shows the wavelength red shift with current due to self-heating which changes the refractive index of the materials in the cavity. In the lower figure, the intensity is normalized with respect to the fundamental mode at each current, where it can be seen that the higher order modes are supressed by more than 38 dB with respect to the fundamental mode.

## Metagrating efficiency estimation details

We use the Fourier plane imaging to estimate the efficiency (**Figure S11**). These images contain background noise from the camera itself, the incoherent LED illumination from the VCSEL and coherent lasing. To correctly estimate the efficiency, we need to remove the former two parts and only evaluate the lasing part. We take 10 images with unbiased VCSEL and biased with 1.4 mA current in a co-polarized and cross-polarized cases. Since the LED part of illumination is incoherent and unpolarized, it appears in both polarization cases. So, we have 4 sets of 10 images, which we average out to eliminate camera shot noise. Then we subtract the background (images with unbiased VCSEL) and are left with two averaged and background-subtracted images: co-polarized and cross-polarized, which we once again take a difference of.


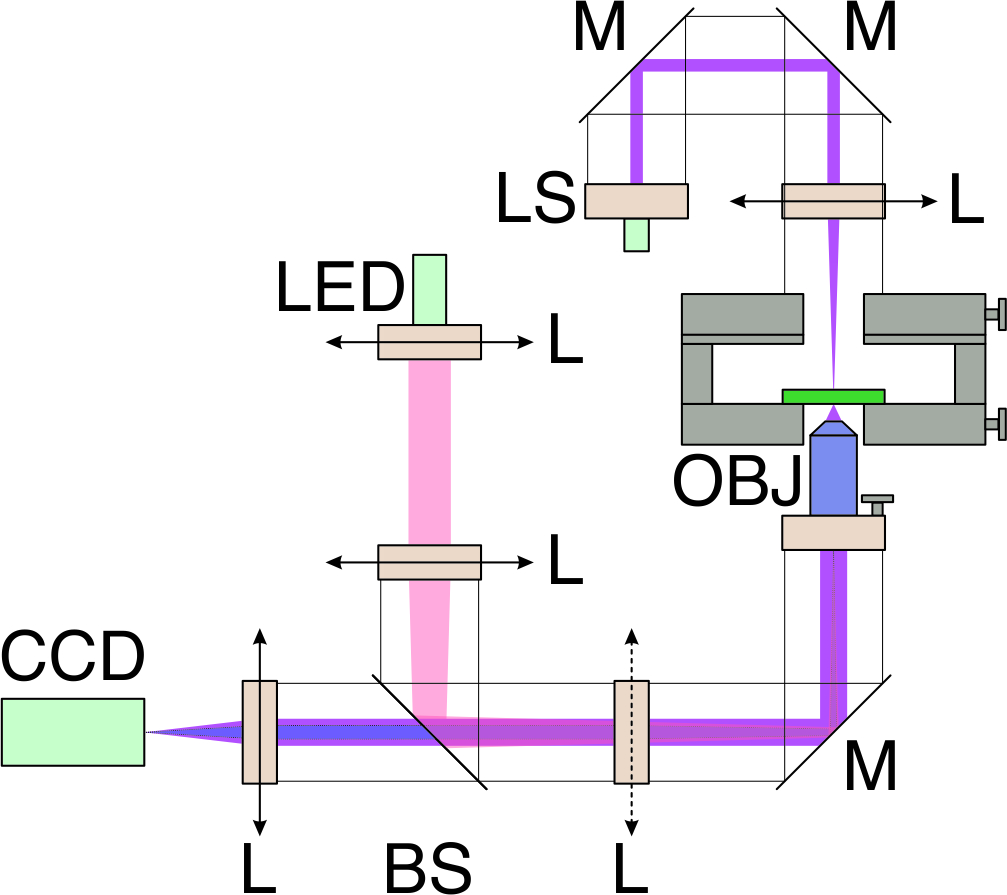


**Figure S11.** Measurement setup: M – mirrors, L – lenses, BS – 50:50 beam splitter, LS – laser source, OBJ – objective lens, LED – incoherent light source, CCD - camera.

Finally, what we are left with is mostly the lasing part. Note that this methodology is imperfect, and some LED and background signals are left in the final image; moreover, the polarizers are imperfect and somewhat distort the images. It is worth mentioning that because of the limited numerical aperture of the collection objective, not all the +1 and -1 order light is captured. All the above skews the results towards less efficient. We evaluate the final efficiency by using 1% threshold of the maximum pixel value (this only selects the +1 order) divided by 0.1% threshold (which selects most of the image and leaves out some of the remaining LED background). This is illustrated in **Figure S12**.


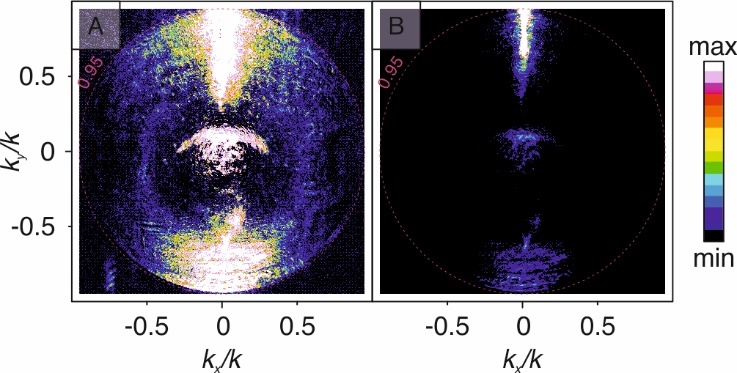


**Figure S12.** Solid white areas mark A) 0.042% (20) threshold to calculate the total intensity and B) 1.053% (500) threshold to calculate the +1 intensity on a background-subtracted, and cross-polarized-subtracted image

To verify the claimed very high efficiency of the metagrating VCSELs we also measured the amount of light in the different diffraction orders in free space with a single photodiode. For each diffraction order the photodiode was placed at the appropriate angle and the VCSEL was biased from the lasing threshold until rollover. The results from the photodetector agreed with the efficiency measurement using the custom microscope setup.


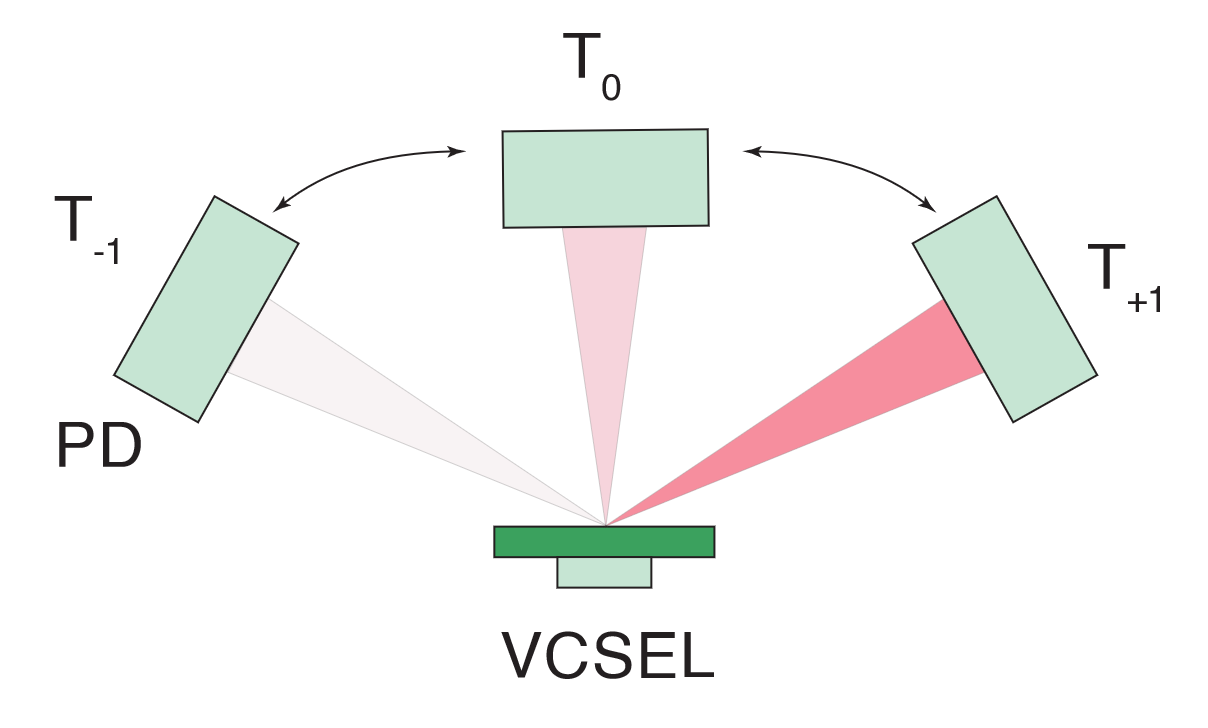


**Figure S13.** Measurement setup: PD – photodetector, T – diffraction orders, VCSEL – laser diode.

## Beam profile measurement

The xz and yz’ beam profiles were measured with a diffusive plate and a CCD camera. The light from the VCSEL illuminates the diffusive plate which is imaged on the camera. To create the beam profiles the imaging system was moved in the z’-direction, tracking the first diffraction order. The beam profile is then put together by stacking the pictures taken every 0.5 mm.


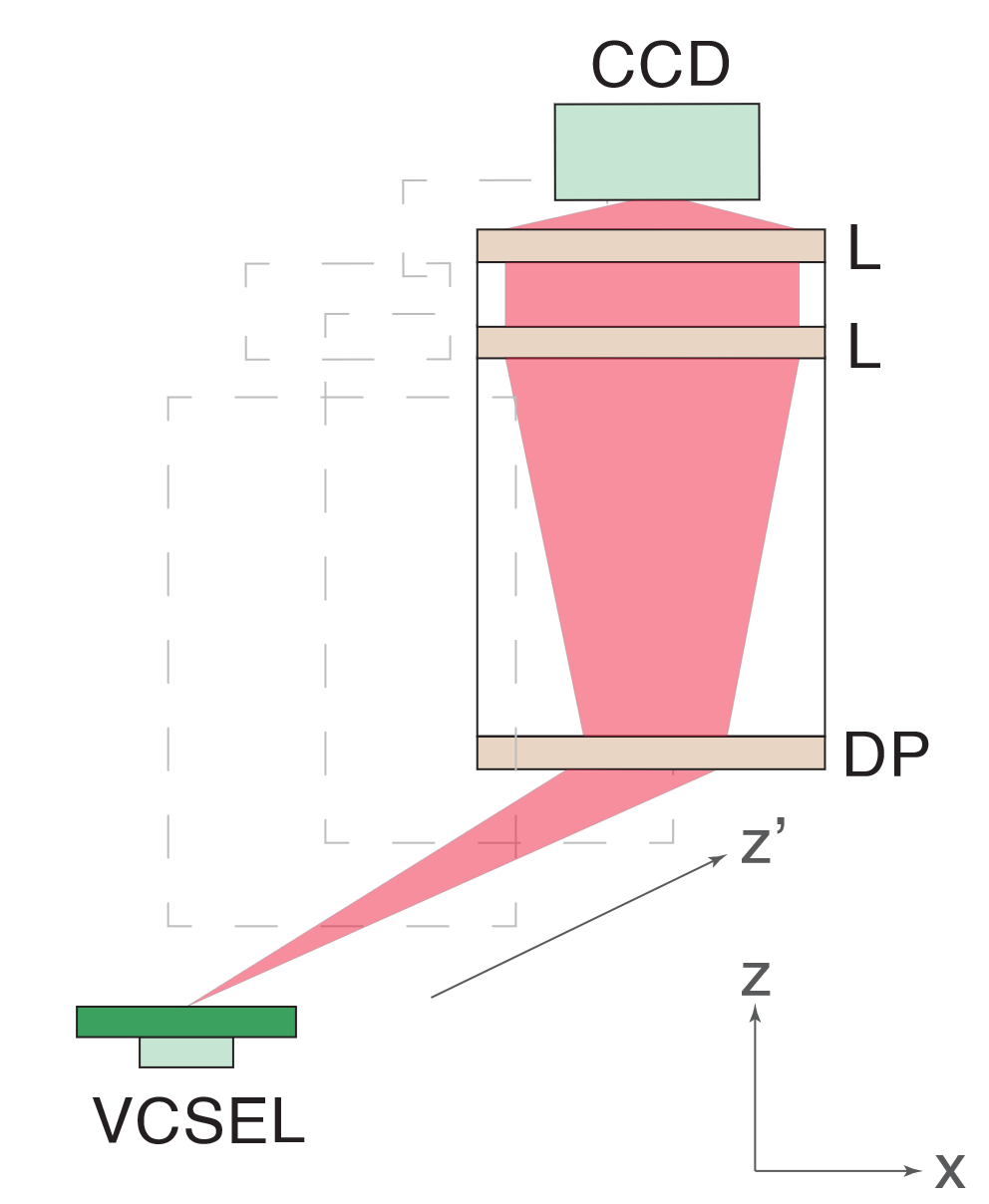


**Figure S14.** Measurement setup: CCD – camera, L – lenses, DP – diffusive plate, VCSEL – laser diode.

## Nanorod synthesis and functionalization

The nanorods (NR) were prepared by seed-mediated growth method using cethydriltrimethylammonium bromide (CTAB) as a stabilizing agent.^1^ The as-synthesized NRs were centrifuged and dispersed in 1 mM CTAB. The NRs were analysed by scanning electron microscopy (SEM) in order to determine size variation. The dimensions of the nanorods were L = 187±12 nm and W = 72.0±6 nm (n=29), respectively.

For the measurements with living cells, the NRs were functionalized with 11-mercaptoundecanoyl-hexaethyleneglycoloic acid (Prochimia, Poland) as follows: Alkanethiols were dissolved in ethanol at a concentration of 10 mM and then mixed with the NR solution to a final concentration of 0.5 mM, corresponding to a CTAB:thiol ratio of approximately 1:4.^2^ The NR solution was incubated in fridge overnight, centrifuged at high speed to remove the supernatant, resuspended in water and finally centrifuged at low speed to remove aggregated nanorods.


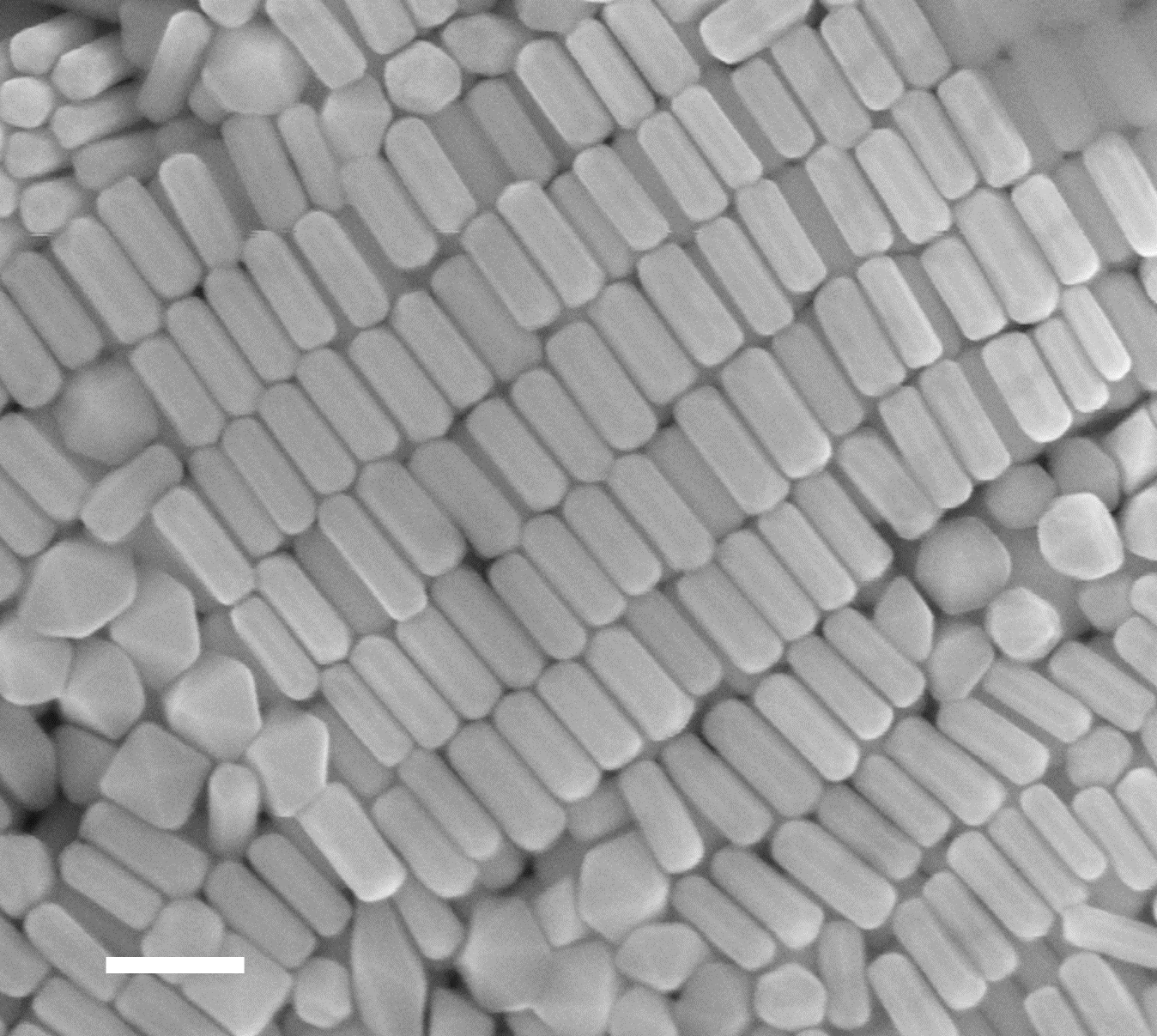


**Figure S15.** SEM image of gold nanorods used in the study. The scale-bar corresponds to 200 nm

## Cell culturing

HMEC-1 cells were cultured at 37°C in MCDB 131 Medium, (Thermo Fisher Scientific, United States) supplemented to a total concentration of 10 mM L-glutamine, 10 ng/mL Epidermal Growth Factor (EGF), 1 ug/mL hydrocortisone, 10% fetal bovine serum (FBS), 1% non-essential amino acids, and 1% penicillin/streptomycin. On the day of the study, cells were harvested by 7 min trypsination (TrypLE™ Express Enzyme (1X), Thermo Fisher Scientific, United States), spun down for 7 min at 200 G whereafter the supernatant was removed, and the pellet was replenished with fresh media and diluted 1:3. Cells were seeded on the cleaned glass cover slips in a culture dish for 2 h. The functionalized nanorods were added and the culture dish was incubated overnight. Fresh culture media without nanorods was replenished just before the measurements.

## References

1. Ye, X.; Zheng, C.; Chen, J.; Gao, Y.; Murray, C. B. Using Binary Surfactant Mixtures To Simultaneously Improve the Dimensional Tunability and Monodispersity in the Seeded Growth of Gold Nanorods. *Nano Letters* **2013**, *13* (2), 765.

2. Kinnear, C.; Dietsch, H.; Clift, M. J. D.; Endes, C.; Rothen-Rutishauser, B.; Petri-Fink, A. Gold Nanorods: Controlling Their Surface Chemistry and Complete Detoxification by a Two-Step Place Exchange. *Angew Chem Int Edit* **2013,** *52* (7), 1934.
